# Phosphorus adsorption using chemical and metal chloride activated biochars: Isotherms, kinetics and mechanism study

**DOI:** 10.1016/j.heliyon.2023.e19830

**Published:** 2023-09-04

**Authors:** Bijoy Biswas, Tawsif Rahman, Manish Sakhakarmy, Hossein Jahromi, Mohamed Eisa, Jonas Baltrusaitis, Jasmeet Lamba, Allen Torbert, Sushil Adhikari

**Affiliations:** a Biosystems Engineering Department, 200 Corley Building, Auburn University, Auburn, AL 36849, USA; b Center for Bioenergy and Bioproducts, 519 Devall Drive, Auburn University, Auburn, AL 36849, USA; c Department of Chemical and Biomolecular Engineering, Lehigh University, USA; d National Soil Dynamics Laboratory, United States Department of Agriculture-Agriculture Research Service, Auburn, AL 36832, USA

**Keywords:** Pine wood, Activated biochar, Eutrophication management, Phosphorus, Adsorption

## Abstract

Efficient treatment of nutrient-rich wastewater is of paramount importance for protecting the ecosystem. In this work, an efficient, abundant, and eco-friendly adsorbent was derived from biochar and employed for phosphorus (P) adsorption. The key factors influencing the P removal efficiency of the activated biochar, including P concentration, pH, dosage, temperature, adsorption time, and influence of co-existing ion type, were investigated. Maximum P adsorption percentage (100%) was obtained with 10 mg/L and zinc chloride activated biochar (BC–Zn) compared to the other activated biochars. Results show that by increasing the P concentration from 5 to 200 mg/L, the phosphorus adsorption capacity increases from 0.13 to 10.4 mg/g biochar. Isotherms and kinetic studies further show that the P adsorption follows the Langmuir and quasi-second-order kinetic models. The mechanistic investigation demonstrated that P adsorption occurred by precipitation reaction. Furthermore, P desorption has been studied at different time intervals to understand the P release rate after adsorption.

## Highlights

• Biochar-derived adsorbents were prepared using different activation methods.
• Higher P removal efficiency (100%) was found with Zn-BC adsorbent.
• The adsorption occurs via the chemical and physical dual processes.
• The P adsorbent has a high potential to be used as a fertilizer.

## Graphical abstract

**Figure:**
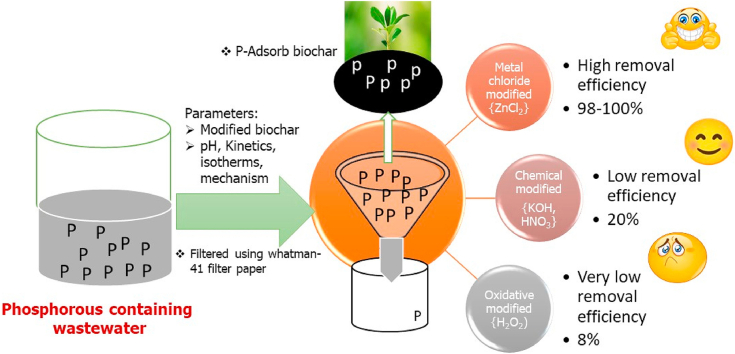

## 1 Introduction

Inadequate use of synthetic and manure fertilizers can lead to excess ammonia nitrogen (N) and phosphorus (P) buildup in soils, lower fertilizer utilization rate, and a higher risk of nutrient loss due to precipitation and irrigation. This problem is exacerbated in areas that are closer to animal production facilities. With the increase in P concentration in the run-off water resulting from fertilizer applications and legacy P, there has been a rapid growth of aquatic plants and algae, leading to eutrophication. Eutrophication has been a threat to the ecosystem of the environment and public health [1,2]. The eutrophication is severe in places, especially where the manure is applied as the source of N. For example, P-rich poultry litter is used as N fertilizer for crops in many parts of the southern states in the US, and as a result, the soil is saturated with P nutrients, which will lead to eutrophication.

Nutrient discharge regulations worldwide are becoming more rigorous to safeguard aquatic ecosystems and public health. The US Environmental Protection Agency (EPA) has established a maximum acceptable limit of 0.1 mg/L of P in treated effluent [3]. In China, the discharge of ammonia nitrogen (N) and P into urban wastewater must not exceed 5 mg/L as NH_4_–N and 0.5 mg TP/L (TP, total P) according to the Grade 1 raised-A standard from GB 18918-2002 [4]. Therefore, a major challenge for sewage wastewater treatment plants (WWTP) today is to remove the excess nutrients from the wastewater in a highly efficient, energy-saving, and cost-effective manner [5]. The purification methods, such as ion exchange, membrane technology, chemical precipitation, biological decomposition, electrochemical technology, and adsorption, have been widely studied for removing P from eutrophic water [6]. Among these technologies, adsorption is the most attractive clean-up strategy for P removal from wastewater because it is cost-effective [7,8]. Recently, the adsorption technology of biochar prepared from waste materials has attracted great attention [9,10]. Particularly, biochar is derived from the residual biomass after the thermochemical process, which can develop economic benefits and reuse the solid waste [11]. Furthermore, the different modification methods of biochar have gained attention due to their cost-effectiveness and can enhance adsorption efficiency. The biochar that had been altered with Ca, Al, and Fe also performed very well in absorbing nutrients. In order to improve soil fertility and trap carbon for an extended period of time, biochar that has been loaded with N or P nutrients can be employed as a slow-release fertilizer [11,12].

Several research groups reported the modification of biochar for P adsorption applications [[13], [14], [15], [16]]. Yin et al. found that the P adsorption capacity significantly increases using Mg-modified (74.47 mg/g), and Al-modified biochar (43.26 mg/g) compared to the raw biochar (3 mg/g). They concluded that the amount, size, morphology, and distribution of Mg and Al oxides in biochar and the formation of chemical bonds between the Mg and P would affect P adsorption [13]. Zamparas et al. used natural bentonite embedded with Fe, Cu, Al ions, and humic acid to remove ammonia nitrogen (N) and P. The authors found maximum P and N adsorption capacity 26.5 mg/g, and 202.1 mg/g, respectively [17]. On the other hand, Jung et al. investigated the P adsorption onto iron-based layered double hydroxides (Fe_3_O_4_@LDHs) adsorbent. The authors found that adsorption mainly depends on the initial pH, P-concentration, and adsorption temperature. Maximum adsorption (36.9 mg/g) was found at 20 °C and pH of 3 [15]. Further, Ren et al. found that the modified ferric oxides/biochar had a reasonably high phosphate adsorption capacity of 0.963 mg/g in comparison to the unmodified cotton stalk biochar (0.0 mg/g), which could barely adsorb phosphate [18]. The advances in terms of maximum removal capacity for the P is excellent. However, these precious metal-loaded adsorbents are hardly deemed cost-effective for P adsorption. Therefore, it is worth trying to incorporate some new metal cations and synthesizing an adsorbent by combining limited air atmosphere calcination would be the more viable for cost as well as the time-consuming process. However, related research on the biochar activation with different chemicals and metal chloride, its calcination under air for a short time, and comparison of different chemical and metal activation processes of biochar for removing nutrients from aquatic environments have rarely been reported.

The overall objective of the study is to develop a versatile biochar-derived adsorbent with different chemical and metal-loaded activation methods under limited air atmosphere calcination and get a higher P removal efficiency from the aqueous media. Different adsorption parameters such as initial pH, adsorbent dosage, contact time, and initial phosphate concentration on the adsorption were investigated. Further, adsorption kinetics and isotherms were determined, and the P adsorption mechanism was elucidated with the help of Fourier transform infrared spectroscopy (FTIR), X-ray diffraction (XRD), and X-ray photoelectron spectroscopy (XPS) analyses. Moreover, the P release kinetics was studied from adsorbent after the P adsorption from the aqueous media.

## 2 Materials and methods

### 2.1 Materials

Pinewood biochar was prepared at Mississippi State University (Starkville, MS, USA) via auger pyrolysis reactor at 450 °C under an N_2_ environment (99.99%) . Biochar was ground into powder form (size between 200 and 400 μm). Metal salts calcium chloride (CaCl_2_.2H_2_O, 99.5%), zinc chloride (ZnCl_2,_ 99%), and potassium phosphate monobasic (KH_2_PO_4,_ 99%) were purchased from the VWR (Radnor, PA, USA) and were used as received. Potassium hydroxide (KOH, ≥85%), nitric acid (HNO_3,_ 70%), hydrogen peroxide (H_2_O_2,_ 30%), potassium nitrate (KNO_3_, 99%), potassium sulfate (K_2_SO_4_, 99%), and potassium chloride (KCl, 99.0%) chemicals were supplied from Sigma Aldrich (St. Louis, MO, USA) and also used as received. P-containing wastewater (WW) samples were collected from a local wastewater treatment plant (H.C. Morgan Water Pollution Control Facility, Auburn, Alabama, USA), and untreated wastewater was used for the adsorption study. The inductively coupled plasma optical emission spectrometry (ICP-OES) results show that WW contains metals such as B (0.89 mg/L), Ba (0.02 mg/L), Ca (23.50 mg/L), Fe (1.01 mg/L), Mg (10.07 mg/L), Mn (0.11 mg/L), Na (15.52 mg/L), S (4.13 mg/L), and Si (8.0 mg/L). Analysis of total carbon (TC) and total nitrogen (TN) showed that the WW had concentrations of 66.85 mg/L-TC and 19.75 mg/L-TN, respectively.

### 2.2 Preparation of modified biochar

#### 2.2.1 Base, acid and oxidative treatment procedure

Pinewood-derived biochar (5 g) was mixed slowly in 2 M KOH (100 mL) solution, and the solution was stirred for 3 h at 40 °C. After that, the biochar solution was dried at 105 °C for 12 h. To avoid the use of excess nitrogen during the calcination and the long time for calcination, here in this study simple and short time calcination process was used. Herein, the dried biochar was wrapped in aluminum foil and then calcined for 5 min under an air environment at 500 °C using a furnace (Thermo Scientific, Inc., Waltham, MA, USA). After the calcination, the calcined biochar was first washed with 1 M HCl solution and then with distilled water several times until the wash water was neutral in pH. The biochar, then, was dried in an oven for 12 h at 105 °C. The biochar obtained after completing these steps was defined as BC-KOH. Further, other activated biochar materials, such as BC-HNO_3_ and BC-H_2_O_2,_ were prepared according to the same process. Unlike the calcined BC-KOH biochar washing process, BC-HNO_3_ and BC-H_2_O_2_ calcined biochar were washed with excess distilled water to make them neutral.

#### 2.2.2 Metal-treatment procedure

First, the pinewood-derived biochar powder (5 g) was added to 100 mL of distilled water and stirred for 30 min. Then the calculated amount of CaCl_2_.2H_2_O (10 wt% Ca) metal salts was added slowly to the biochar solution. After that, the solution was stirred for 3 h at 40 °C. Then, the biochar and metal salts solution were kept in an oven at 105 °C for 12 h to dry. Then, the dried biochar was calcined at 500 °C using a furnace for 5 min under an air environment. After the calcination, calcined biochar was washed using water several times to remove excess chlorine. The biochar was dried at 105 °C for 12 h and was defined as BC-Ca. The same procedure was used for the preparation of other metal ions biochar, such as 10 wt% Zn using ZnCl_2_ and 5 wt% each Ca and Zn using respective chloride salts -hereafter referred to as BC-Zn and BC-Ca/Zn.

### 2.3 Adsorption experiments

Adsorption of phosphorus (P) was carried out using batch methods at 25 °C at different pHs of 2–11. A known amount (0.5 g) of biochar and 50 mL of P solution with various concentrations (5–200 mg/L) were added in 100 mL conical flasks and shaken at 150 rpm. The initial pH of the biochar and P mixture solution was measured using a pH meter for each experiment. The pH of the solution was adjusted with 0.1 mol/L NaOH and HCl solution. After shaking for 24 h, the mixture was filtered through a Whatman-40 ashless filter paper. The P concentrations of the filtrates were determined with the help of molybdovanadate using the acid persulfate digestion method (Hach Method, Instrument DR 900, Range: 0.0–100.0 mg/L PO4, Hach, Loveland, Colorado, USA). In this method, a sample was first treated with potassium persulfate to convert any phosphorus present into orthophosphate ions. Then, the sample was treated with molybdovanadate, which forms a complex with the orthophosphate ions. The formation of this complex is proportional to the concentration of phosphorus in the sample, and the intensity of the color produced can be measured to determine the concentration of phosphorus. Duplicate experiments were mainly performed for higher and lower adsorption experimental group to assure that the experimental deviation and it was within ±5%. The adsorption capacity Q_t_ (mg/g) and removal rate of the P were calculated using equations (1) and (2), respectively.

(1) $$\text{Adsorption}\;\text{capacity}\;\left(Q_{t}\right)=\frac{\left(C_{0}-C_{e}\right)}{m}\times V_{L}$$

(2) $$\text{Removal}\;\text{rate}=\left(1-\text{Ce}/C_{0}\right)\times 100\%$$

where C_0_ and Ce (mg/L) are the initial and equilibrium P concentrations, respectively; V_L_ is the solution volume (L); and m is the mass of the biochar (g).

### 2.4 Isotherms and kinetics of P adsorption

Adsorption isotherm experiments were carried out at various P concentrations ranging from 5 to 200 mg/L. For this, each conical flask consisted of 50 mL P solution, to which 0.5 g modified biochar adsorbent was added. Then, the equilibrium phase was obtained after stirring the solution in the flask for 24 h. After the equilibrium, the mixture was filtered through a Whatman-40 ashless filter paper. The P concentrations in the filtrates were determined as discussed in Section 2.3. The adsorption capacity of the selected biochar for P was determined with the commonly used isotherm models such as Langmuir, Freundlich, and Temkin. The models are expressed by equations (3), (4).

(3) $$\frac{\text{Ce}}{\text{Qe}}=\frac{\text{Ce}}{\text{Qm}}+\frac{1}{K_{L}.\;\text{Qm}}\;\text{Langmuir}$$

(4) $$\ln\text{Qe}=\ln\;K_{F}+\frac{1}{n}\ln\text{Ce}\;\text{Freundlich}$$

and

(5) $$Qe=\frac{RT}{b_{T}}\;\left(K_{T}Ce\right)\;\text{Temkin}$$

where *Q*e is the amount adsorbed at the equilibrium (mg/g) and *Q*_m_ is the theoretical maximum adsorption capacity (mg/g). *C*e, K_L,_ K_F_, b_T_, K_T_, T and R represent the equilibrium concentration, Langmuir equilibrium sorption constant (0.1600 L/mg), Freundlich equilibrium sorption constant (2.180 mg^1-1/n^.L^1/n^.g^−1^), Temkin constant to the heat of adsorption (1.903 J/mol), equilibrium binding constant (1.485 L/mg), absolute temperature (K), and the gas constant (8.314 J/mol K), respectively.

The adsorption kinetics of the biochar was evaluated using 100 mL conical flasks consisting of 50 mL of a 100 mg/L P solution and 0.5 g adsorbent. The initial pH (pHi) of the batch experiment was set to 9 ± 0.2. The pH value was maintained with the help of either 0.1 mol/L HCl or 0.1 mol/L NaOH. Then, the flasks were sealed and stirred at 25 °C for 6, 12, 18, and 24 h at a rate of 150 rpm to reach adsorption equilibrium. After the desired time, the solution was filtered, and the concentration of P was measured. Mathematical pseudo-first-order and pseudo-second-order models were used to determine the contact time for P adsorption by non-linear regression. The models are given by equations (6), (7)):

(6) $$\ln\;Q_{e}-Q_{t}=\ln\;Qe-K_{1}t\;\text{First}-\text{order}$$

(7) $$\frac{t}{Q_{t}}-\frac{t}{Q_{e}}=\frac{1}{K_{2}Q_{e}^{2}}\;\text{Second}-\text{order}$$

where, K_1_ and K_2_ are the equilibrium rate constant of the pseudo-first-order equation (1/h), the pseudo-second-order rate constant g/(mg^1/2^ h) and t is the contact time (h); the parameter Q_t_ is the adsorption amount (adsorption capacity) at a certain time (mg/g); and Q_e_ is the adsorbed amount at equilibrium (mg/g).

### 2.5 Characterization methods

Thermogravimetric analysis (TG) was conducted using a TGA-50 thermogravimetric analyzer (Shimadzu, Columbia, Maryland, USA) at the heating rate of 10 °C/min from the room temperature to 800 °C in an N_2_ atmosphere. The specific surface area, pore, and pore volume of adsorbents were estimated by using Autosorb-iQ (Quantchrome Instruments, Boynton Beach, FL, USA), and the isotherms were analyzed at −196.15 °C under liquid N_2_. Fourier Transform Infrared Spectroscopy (FTIR, Thermo Scientific Nicolet 6700, Waltham, Massachusetts, USA) was performed to analyze the functional groups of the biochar samples. The pH of the solutions was determined by a pH meter (Okaton, series 510, Wilmington, NC, USA) [12]. Elements (CHNS) present in samples were analyzed using a vairo macro cube elemental analyzer unit (Ronkonkoma, NY, USA), while oxygen was calculated by the difference. The point of zero charge (pH_PZC_) was determined with the help of the method discussed in the published literature [13]. The XRD patterns were acquired using an Empyrean, PANalytical B.V. diffractometer (Westborough, MA, USA). The diffraction patterns were obtained between 5 and 70°. The ex-situ mode was utilized to collect the XPS spectra in a SPECS Near Atmospheric Pressure X-ray Photoelectron Spectroscopy (NAP-XPS) system. This system comes equipped with an XR 50 MF Al Kα X-ray Source, utilizing a μ-FOCUS 600 X-ray monochromator with a pass energy of 20 eV and 100 W power. Spectral data were processed using the CasaXPS software suite [19].

## 3 Results and discussion

### 3.1 Analysis of adsorbent

The Brunauer-Emmett-Teller (BET) specific surface area, total pore volume, and average pore diameter of a different adsorbent, such as raw biochar, and BC-Zn, are presented in Table 1. The specific surface area of raw biochar is very low (1.10 m^2^/g) compared to the BC-Zn modified (4.11 m^2^/g) and after P adsorption, BC-Zn-P, biochar (26.04 m^2^/g). It may be due to the lower undeveloped porous structure in the raw biochar, while after activation, the biochar porous structure was developed. Moreover, calcination at higher temperatures promoted Zn-bounded hydroxide or moisture release from the biochar surface, which helped to create a higher porous structure [20]. A higher surface area for the P-adsorbed BC-Zn biochar can be attributed to the basic solution (adjust pH to 9) during the adsorption experiment cleaning the surface by removing the impurities. BC-Zn-activated biochar and after P adsorption biochar (BC–Zn–P) had lower pore diameters (1.71 nm and 1.73 nm, respectively) and higher pore volumes (0.006 cc/g and 0.025 cc/g, respectively) compared to raw biochar (1.91 nm and 0.005 cc/g). From the BET analysis result, it is confirmed that all biochars are corresponding to a mesoporous structure.

**Table 1:** Surface area, Pore volume and diameter of modified biochar and after p adsorption biochar.

Sample	Surface area, m^2^/g	Pore volume, cc/g	Pore diameter, nm
BC	1.10	0.005	1.91
BC-Zn	4.11	0.006	1.71
BC-Zn-P^a^	26.04	0.025	1.73

a After P adsorption.

The elemental compositions of biochars are presented in Table 2. In comparison to the raw biochar carbon content (80.1%), the carbon content for chemically-modified biochar (67.90–75.03%) and metal chloride-modified biochar (71.16–79.24%) decreased sharply with the increase in oxygen content. This may be due to the oxidative calcination of modified biochar, where some carbon is lost in the form of CO and CO_2_, and oxygen is impregnated with the biochar [21]. The ratios of H/C and O/C in biochar can be correlated to aromaticity, hydrophilicity, and polarity. Decreasing the H/C ratio increases the aromaticity of biochar, while increasing the O/C ratio indicates an increase in the hydrophilicity and polarity of the biochar [22]. Thus, when KOH and ZnCl_2_ are used to modify biochar, aromaticity gradually decreases while there is an increment in the hydrophilicity and polarity progressively. Whereas higher H/C and O/C ratios in the modified biochar indicate that more oxygenated functional groups are formed [23], which was also confirmed by the FTIR analysis. After the adsorption of P on BC-Zn biochar, hydrogen content increased, which indicates that BC-Zn adsorbent effectively adsorbs P in the form of HPO_4_^2−^ from the aqueous phase.

**Table 2:** Elemental distribution of different modified biochars, and after P adsorption biochars wt.%.

Samples	C	N	H	S	O^a^	O/C	H/C
BC	80.1	0.50	3.47	1.58	12.37	0.12	0.53
BC-KOH	67.90	0.59	2.93	1.04	25.95	0.29	0.52
BC-HNO_3_	73.48	3.58	2.68	0.98	18.05	0.17	0.44
BC-H_2_O_2_	75.03	0.55	3.19	0.63	19.25	0.19	0.51
BC-Ca	79.24	0.59	3.37	0.70	13.73	0.14	0.51
BC-Zn	71.16	0.46	2.92	0.36	22.27	0.25	0.49
BC-Ca/Zn	74.36	0.30	3.09	0.36	19.25	0.21	0.50
BC-P^b^	68.97	0.35	3.07	0.23	25.61	0.27	0.53
BC-Zn-P^b^	74.37	0.37	3.15	0.64	19.74	0.21	0.51

a Calculated by the difference (O% = 100- (C + H + N + S + Ash).

b After P adsorption.

**Fig. 1:**
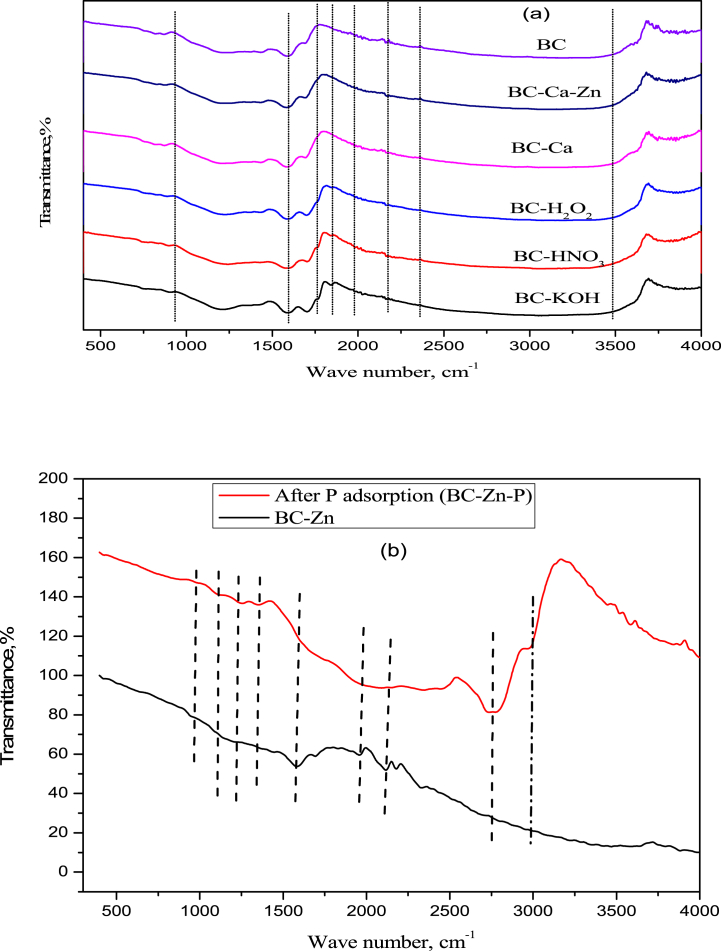
FT-IR spectra of (a) raw biochar, modified biochars obtained by different methods, and (b) BC-Zn modified biochar and after P adsorption biochar (BC–Zn–P).

The ratios of the peak height of diamond and graphite carbon, such as the I_D_/I_G_ ratio, are commonly employed to evaluate the level of defects in carbon materials, where a higher I_D_/I_G_ value indicates a greater presence of functional groups with defects in the material (Figure S1). The I_D_/I_G_ intensity ratio of BC, BC-Zn, and BC-Zn-P are 0.71, 0.42, and 0.31, respectively, showing that BC-Zn and BC-Zn-P have a higher content of graphite carbon [20]. It is implied that the activation of biochar by using ZnCl_2_ enhanced the graphitization of the biochar. FTIR spectra of different adsorbents and after P adsorption (BC–Zn–P) are analyzed to understand the changes of different functionality during biochar modification, as shown in Fig. 1a and b. The peaks present at 3695 cm^−1^ are due to OH, which can be attributed to the Zn–OH stretching [20]. The peaks present at 3427 cm^−1^ are assigned to O–H stretching vibrations of water molecules, while the peaks present at 1615 cm^−1^ are assigned to the H–*O*–H stretching vibrations and bending vibrations of hydroxyl functional groups [24]. Stretching frequencies of aromatic C=C are represented by the peaks at 1528 cm^−1,^ and peaks at 1430 cm^−1^ can be attributed to the vibrations of carboxyl groups (-COOH) (Fig. 1a). The peaks at 1069 cm^−1^ are attributed to C–O stretching [25]. The peaks at 853 cm^−1^ and 776 cm^−1^ can be ascribed to the stretching vibrations occurring in the P–O bond and P=O bond, respectively [24]. The peaks at 559 cm^−1^ are due to the lattice vibrations of P–O bonds [26]. Stronger adsorption peaks of P-adsorption adsorbent (BC–Zn–P) indicated that BC-Zn successfully adsorbs the P on it (Fig. 1b), which is associated with the increased values of hydrogen also. After P adsorption, new peaks of Zn–P–O and P–OH were obtained at lower wavenumber. P–*O*–P stretching bridge and asymmetric vibration peak were observed at 556 cm^−1^ and 1109 cm^−1^ [24,26]. Moreover, the peak at 629 cm^−1^ confirmed that Zn–*O*–P stretching vibration, which indicated that P adsorbed onto the Zn-doped biochar by chemical bond formation.

**Fig. 2:**
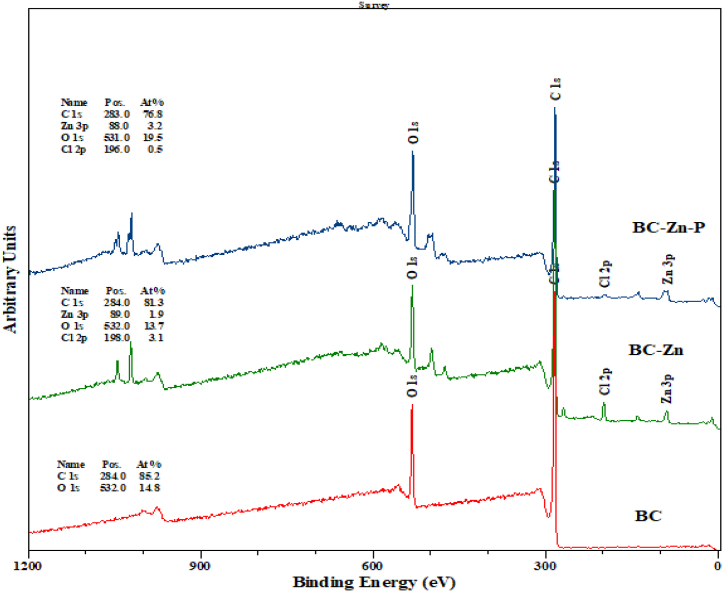
XPS analysis of raw biochar, BC-Zn and after P adsorption biochar (BC–Zn–P).

The appearance of phosphorus adsorption peaks in the XPS spectra confirms that the ZnCl_2_ activation introduces zinc-containing groups into carbon-based materials (Fig. 2). In particular, peaks due to the Zn 2p and Cl 2p are present in the survey XPS spectra as a doublet at 1022 eV and 200 eV, while observation of P content on the sample surface was inhibited by the overlapping Zn 2p and Cl2p peaks. XPS-derived quantification showed that BC contained 85.2% carbon and 14.8% oxygen, while BC-Zn contained ∼2% Zn and 3% Cl. BC-Zn-P contained only 0.5% Cl.

XRD patterns of the BC, BC-Zn, and BC-Zn-P show that there were new peaks that appeared after the activation of biochar (BC–Zn) and after P adsorption (BC–Zn–P) (Figure S2). Peaks corresponding to Zn were obtained at 11.2° due to the ZnCl_2_ present activated biochar. On the other hand, P adsorption biochar (BC–Zn–P) peaks slightly shifted to 9.5°, which might be due to the phase change of Zn metal after P adsorption. However, after P adsorption onto the BC-Zn adsorbent, several new peaks of Zn–P, Zn–O, or ZnHPO_4_ were pointed at 19.5°, 31.2°, 45.3°^,^ and 46.8°. . The different raw biochars, BC-Zn, and P adsorption onto the BC-Zn were shown different thermal stabilities. From TGA results (Figure S3), it has been shown that the raw biochar began to decompose at higher temperatures, which illustrates its strong thermal stability. Significant weight loss of all the biochar was observed at the temperature ranges from 350 to 550 °C. However, the rate of weight loss was higher for the modified biochar (BC–Zn) and P-adsorbed biochar (BC–Zn–P), and also the temperature at which the rate of mass loss was maximum (T_max_) shifted from 507 °C (for BC) to 474 °C for the modified and P absorbed biochar samples (BC–Zn and BC-Zn-P). It is interesting to note that the final weight of BC-Zn-P was much lower than BC-Zn. This suggests that BC-Zn-P biochar was the most unstable among the three samples and promoted carbon decomposition reactions.

### 3.2 Adsorption study

#### 3.2.1 Effects of different adsorbents on P adsorption

**Fig. 3:**
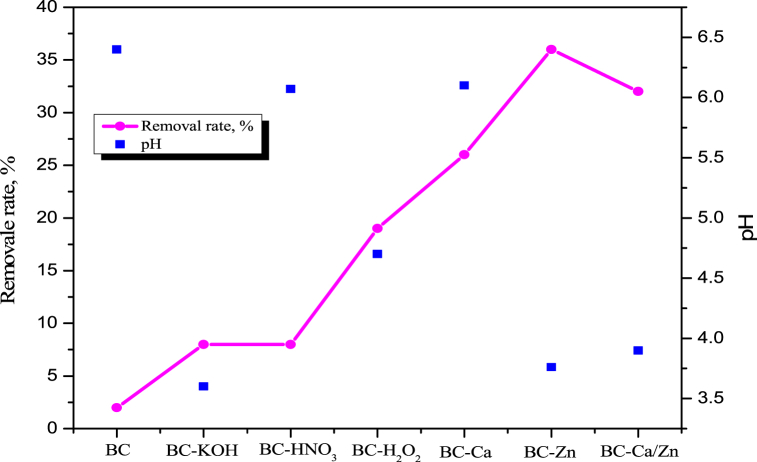
Effects of different modification biochar type on P adsorption.

The effects of different adsorbents on P adsorption are shown in Fig. 3. From Fig. 3, it is observed that adsorbents such as BC, BC-KOH, BC-HNO_3_, BC-H_2_O_2_, BC-Ca, BC-Ca/Zn, and BC-Zn showed the removal percentage of 2.0, 8.0, 8.0, 19.0, 26.0, 32.0, 26.0, 32.0, and 36.0%, respectively. The P removal efficiency of the raw and chemical activation biochars was very low, with a value for removal rate of only 2–19%, which can be attributed to the hindrance of the affinity for binding due to electrostatic repulsion between the negatively charged surface of the biochars and phosphate anions [27]. However, the case of biochar modified with metals such as CaCl_2_, ZnCl_2_, and a mixture of CaCl_2_–ZnCl_2_ showed better P removal efficiency. This might be due to the positive charge that metals have, which provides the active sites for the electrostatic attraction and results in a higher removal rate [6]. The initial pH (pH_i_) of each solution showed that with the addition of Zn metal chloride, the pH of the solution changed, and a lower pHi solution (3.76 ± 0.02) was found in the case of the BC-Zn solution. While the pHi value of BC-Zn biochar is lower than the point of zero charges (PZC) 3.85 (Figure S4). If the pH of the solution is lower than the PZC value, the biochar consists of a positively charged surface whereas, if it is higher than the PZC value, it will consist of a negatively charged surface [28]. Among the metal chloride-modified biochar, the highest P removal rate (36%) was observed with the ZnCl_2_-modified biochar (BC–Zn). This may be due to the lower pH (3.76 ± 0.02) value of the BC-Zn phosphorus solution, which produced more positively charged (Zn^2+^) surfaces that promoted the electrostatic attraction between the phosphate anions [29]. Further, Zn^2+^ ions have promoted chemical precipitation to P removal percentage from the aqueous phase. Li et al., 2019 research group showed that with the addition of Ca and Mg salts during the P adsorption, metal ions commonly engage in precipitation reaction and form Ca_5_(OH)(PO_4_)_3_ (hydroxyapatite HAP) and MgNH_4_PO_4_.6H_2_O, respectively [24,30]. Sole biochar as an adsorbent participated mainly by electrostatic interaction, while metal-biochar employed both electrostatic attractions as well as the precipitation complex reaction with P, resulting in the enhanced P removal efficiency from the aqueous phase. Several research groups investigated P adsorption onto various modified biochar [27,31]. The authors found the maximum adsorption capacity of 3.08 mg/g with α-Fe_2_O_3_/Fe_3_O_4_ modified bamboo biochar and 3.2 mg/g with FeCl_3_-modified pinewood sawdust biochar, which was a much lower adsorption capacity compared to this study.

#### 3.2.2 Effect of pH and adsorbent dosage on P adsorption

**Fig. 4:**
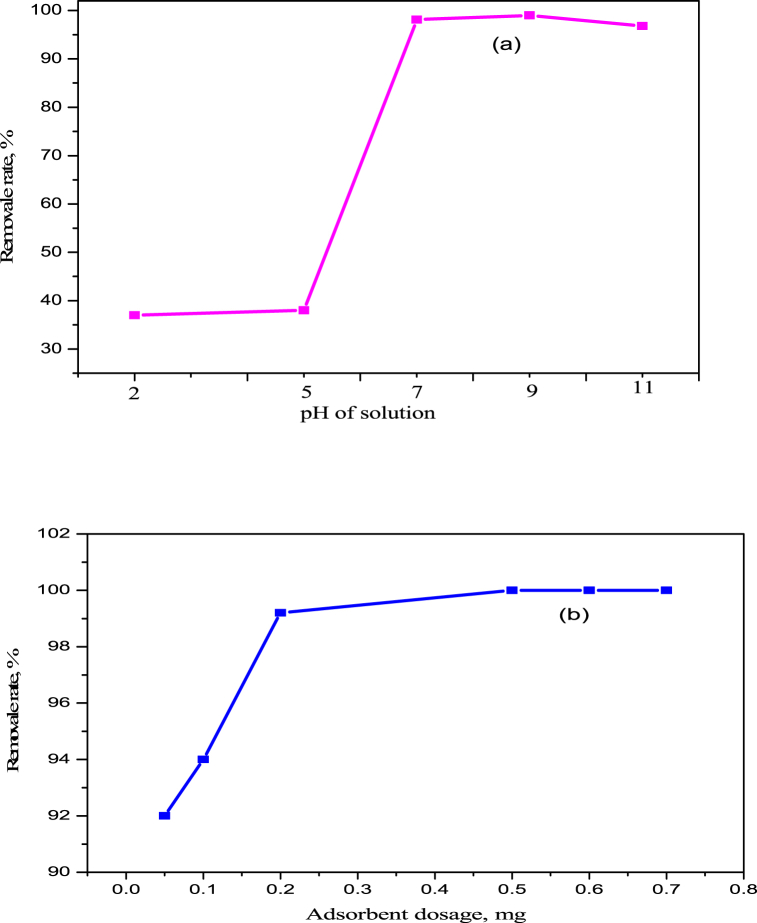
Effects of (a) pH, (Obtained with 0.5 g adsorbent, 50 mL 10 ppm P solution and pH adjust to 9.0 for 24 h time at 25 °C) and (b) adsorbent dosages on the removal rate of P using BC-Zn modified biochar, (Obtained with different adsorbent amount, 50 mL 10 ppm P solution and pH adjust to 9.0 for 24 h time at 25 °C).

Fig. 4a shows the effect of initial pH on the P adsorption. It was observed that with the increase in pH from 2 to 9, the adsorption increased from 37% to 99%. With the further increase in pH from 9 to 11, the adsorption decreased from 99.02% to 96.8%. Maximum adsorption of 99.02 of P was obtained at pH 9 (basic range). Phosphate present in a solution goes through a series of transformations with changes in pH, resulting in the formation of H_2_PO_4_^−^ (pH 2.13–7.20), HPO_4_^2−^ (pH 7.20–12.33), and PO_4_^3−^ (pH ≥ 12.33), as reported by Xie et al. (2013). Of the three forms, HPO_4_^2−^, PO_4_^3−^, and H_2_PO_4_^−^, the adsorption capacity followed the order of HPO_4_^2−^ > PO_4_^3−^ > H_2_PO_4_^−^, with a greater tendency for adsorption on the adsorbent surface. Higher adsorption of P at pH 9.0 was mainly due to the complexion and precipitation reaction between the HPO_4_^2−^ and Zn^2+,^ which could form a crystal of ZnHPO_4_. The chemical precipitation of P on the BC-Zn was further confirmed by XPS analysis. The Zn2p spectrum demonstrated that three compounds were presented on the P adsorbent surface. The binding energy at 347.5 eV corresponds to ZnHPO_4_. Further increasing the pH from 9 to 11, the adsorption decreased to 96.8%, it might be due to the functional groups that consist of oxygen creating more negative charge on the adsorbent surface, leading to the repulsion between adsorbent and PO_4_^3−^ thereby reducing the adsorption [24,32]. This result demonstrated that the phosphate present at lower pH, such as in the form of H_3_PO_4_, and H_2_PO_4_^−^ are not capable of participating in chemical complex reactions and precipitate with the Zn^2+^, while it occurred at higher pH between the phosphate form of HPO_4_^2−^ and Zn^2+^. Whereas in the case of only biochar as an adsorbent, the adsorption was low, 38% compared to the Zn metal-supported biochar. A similar result was also found by Deng et al., they obtained higher adsorption at a pH range from 7 to 11 with Ca^2+^ and Mg^2+^ supported biochar compared to the nonmetal marble waste (Mar-BC800) and calcium-rich sepiolite (Sep-BC800) biochar [24].

Adsorbent dosage is an important parameter for the adsorption of phosphorus. The removal percentage of phosphorous at different dosages is shown in Fig. 4b. With the increase in the amount of BC-Zn adsorbent from 0.05 g to 0.5 g, the P removal % increased from 92.0% to 100.0%. Further, increasing the adsorbent amount, the removal percentage remained constant. This is because of the increase in the specific surface area, active sites, and Zn^2+^ ions for the adsorption of P with the increase in the adsorbent amount [30]. From the perspective of economic cost, 0.5 g adsorbent (BC–Zn) was considered the optimal dosage for P adsorption.

In addition, in this study P containing wastewater (WW) was used to understand the adsorption of P, and the same adsorption study parameters were used as mentioned experimental section. The WW had a P concentration of 1.3 mg/L, and the results showed that BC-Zn adsorbent was able to adsorb 100% of the P from the wastewater. The P adsorption will be highly dependent on its initial concentration and the amount of biochar used for the adsorption.

#### 3.2.3 Effect of Co-existing element on P adsorption

Different competing ions such as SO_4_^−2^, Cl^−^, and NO_3_^−^ are present in the wastewater, which would affect the actual P adsorption by BC-Zn adsorbent. Thus different ions solution (10 mg/L) was prepared using KNO_3_, K_2_SO_4_, and KCl salts, and mixed with the P solution. The effect of P adsorption in the presence of different competing ions was investigated. The co-existing cations at a low concentration (10 mg/L) had very less impact on P removal using BC-Zn adsorbent (Figure S5). The mixture of co-existing ions showed low adsorption of P, and it might be due to the anion with a large radius and high valence (SO_4_^−2^, and NO_3_^−^) being easily captured by adsorbents. Due to this, active sites of adsorbent are blocked, resulting in the reduction of the adsorption efficiency of the adsorbent. Whereas, the presence of Cl^−^ anion alone showed a 1% higher adsorption compared to the other ions. It might be due to the Cl^−^ forming stable complexes with the phosphate and remaining in the adsorbent.

#### 3.2.4 Effect of P concertation on adsorption and isotherms

**Fig. 5:**
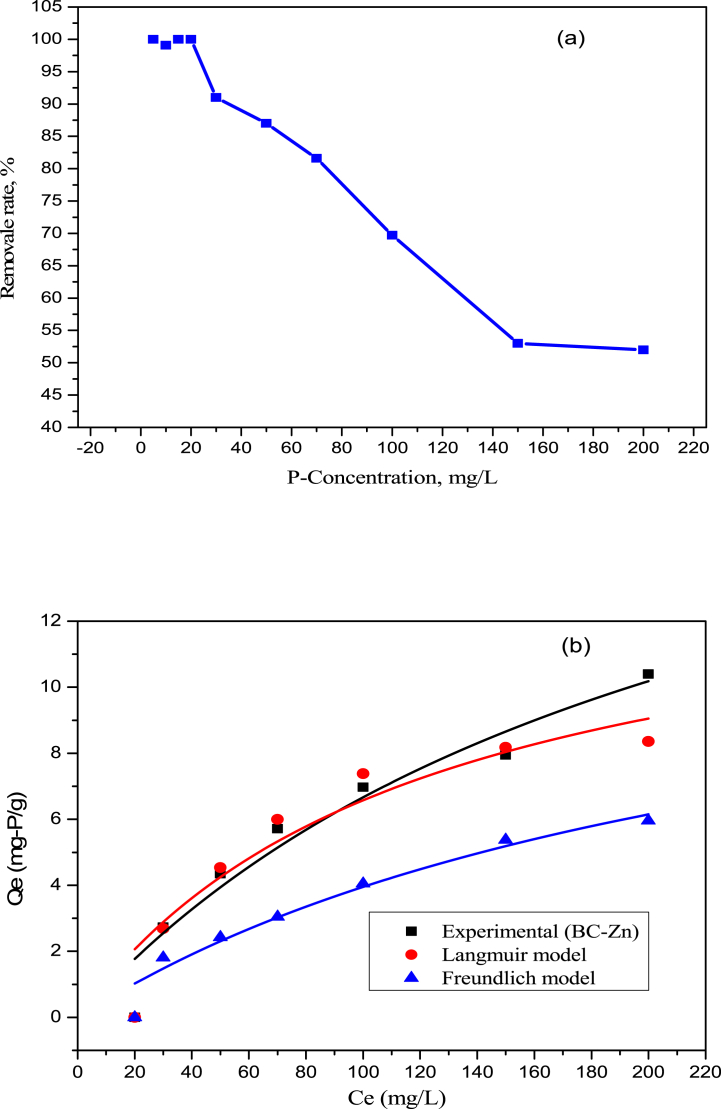
(a) Effects of initial P concentration on P removal rate and (b) adsorption isotherms study, (Obtained with 0.5 g adsorbent, 50 mL different P solution and pH adjust to 9.0 for 24 h time at 25 °C).

The effect of concentration on the adsorption is shown in Fig. 5a. The adsorption was observed to be 100% for the concentrations of 5, 10, 15, 20, and 30 ppm. During the increase in P concentration from 30 ppm to 150 ppm, the adsorption decreased rapidly from 100% to 53%. With the further increase in the concentration from 150 ppm to 200 ppm, the adsorption decreased to some extent from 53.0% to 52.0%. It indicates that the active site of the adsorbent is blocked by the phosphate anion and further could not adsorb P onto the adsorbent. Adsorption isotherms were analyzed using the initial 100 ppm P solution with 0.5 g BC-Zn adsorbent, and the results are shown in Table 3. Fig. 5b presents the P adsorption isotherms of experimental, Langmuir, and Freundlich models, and parameters that are shown in Table 3. From the table, it can be seen that the R^2^ value of the Langmuir isotherm model (0.97) is higher compared to the Freundlich isotherm (0.94) and Temkin isotherm model value (0.93). Furthermore, the Langmuir isotherm adsorption capacity value (8.9 mg/g) is close to the experimental value (10.4 mg/g). This indicates that monolayer adsorption occurred during the P adsorption onto the BC-Zn adsorbent. However, multilayer adsorption has also been processed, which is confined to the Freundlich R^2^ value and the pseudo-second-order kinetics model data. Although the adsorption capacity (5.95 mg/g) for the Freundlich isotherm was very low compared to the experimental data. Overall, BC-Zn biochar showed an efficient P-removal percentage compared to the other research groups' P-removal results.

**Table 3:** Summary of parameters derived from first and second-order reaction kinetics.

Adsorption kinetics											
Pseudo-first-order			Pseudo-second-order								
K_1_	Q_e_(mg/g)	R^2^	K_2_	Q_e_ (mg/g)	R^2^						
−0.0047	5.631	0.78	0.048	7.44	0.93						
**Adsorption Isotherms**											
**Langmuir model**				**Freundlich model**					**Temkin Isotherm**		
K_L_	R_L_	Q_e_	R^2^	K_F_	n	Q_e_	R^2^		BT(jmol-1)	KT(L mg^−1^)	R^2^
0.160	0.058	8.9	0.97	2.180	2.99	5.95		0.94	1.903	1.485	0.93

Q_e_ (mg/g), k_1_ (1/min), k_2_ g/(mg.min), kp (mg/(min^1/2^ g)), Q_m_ (mg/g), k_L_ (L/mg), k_F_ ((mg/g).(L/mg)^1/n^). Experimental Q_e_ = 10.4 mg/g.

#### 3.2.5 Effect of time and kinetics on P adsorption

**Fig. 6:**
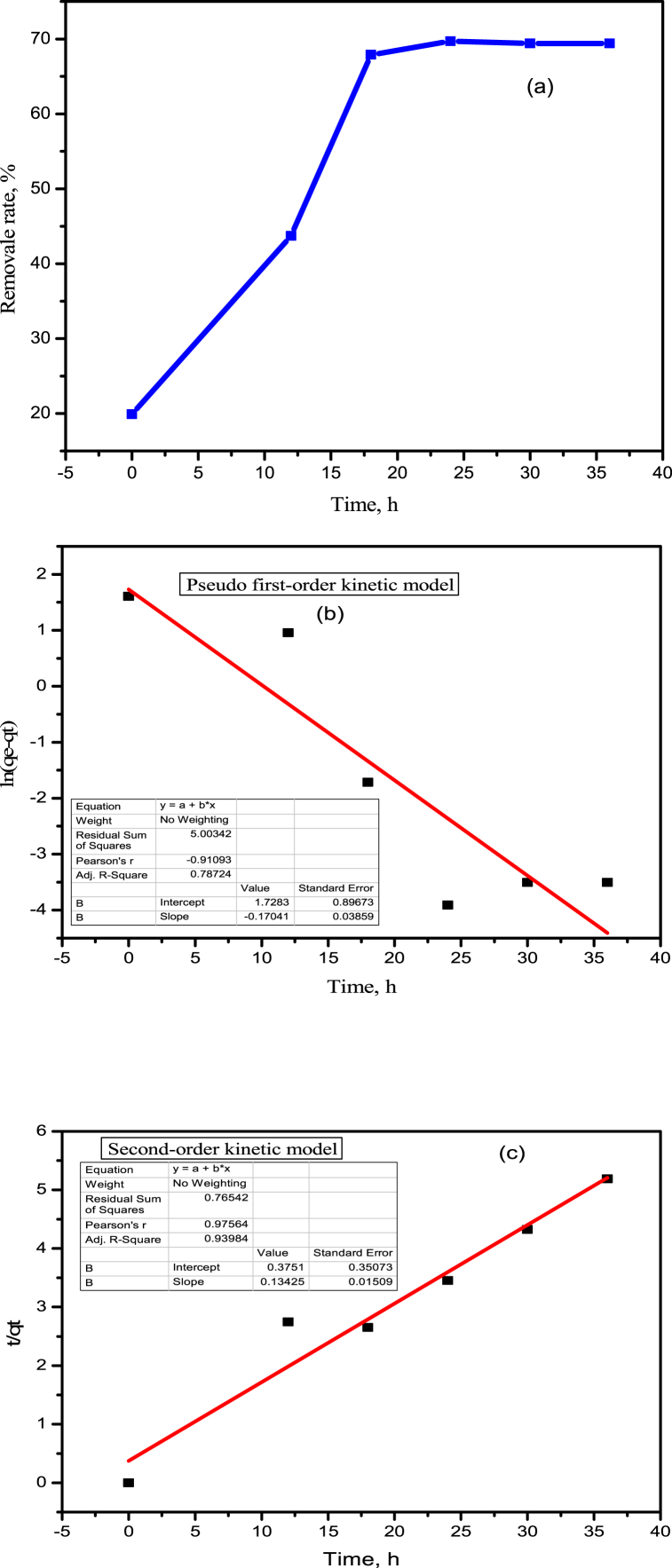
(a) Effect of time on P adsorption and (b) First order kinetics analysis and (c) Second order kinetics analysis on P adsorption, (Obtained with 0.5 g adsorbent, 50 mL 100 ppm P solution and pH adjust to 9.0 for 24 h time at 25 °C).

Fig. 6a shows the effect of time on the adsorption of P. With the increase in time, there was an increase in adsorption. Specifically, when the time increased from 0 h to 24 h, the adsorption increased from 19.9% to 69.7%. After 24 h, with the increase in time to 30 and 36 h, the adsorption efficacy remained the same.

Kinetics plays an important role in the adsorption behavior and can greatly influence the adsorption rate, equilibrium time, desorption rate, and prediction of the adsorption behavior. Therefore, an understanding of the kinetics of the adsorption process can lead to more efficient and effective treatment methods for removing pollutants from wastewater. The adsorption kinetic of BC-Zn adsorbent was described using a pseudo-first-order and pseudo-second-order kinetic model, and fitting best-fit parameters are shown in Table 3 and Fig. 6b and c. The P adsorption rate correlation coefficient value (R^2^) of the pseudo-second-order model, as shown in Fig. 6b, was as high as 0.93, which is greater than that of the pseudo-first-order (0.78). Moreover, the calculated Q_e_ from the pseudo-second-order (7.4 mg/g) is almost closer to the experimental result (6.97 mg/g). This demonstrates that the pseudo-second-order model is adequate to explain the phosphate adsorption on BC-Zn biochar. This also indicates that chemisorption is the rate-controlling step (dominant) for P adsorption, which is consistent with the results from Yin et al., [33]. Meanwhile, the pseudo-first-order model cannot pass through the origin and has a high intercept value, which indicates that the adsorption is controlled by multiple processes. This result is different from some studies [24,29], which indicate that surface active sites on BC-Zn have a major role during the P adsorption process.

#### 3.2.6 Thermodynamic analysis

Thermodynamic properties of the adsorption process can change due to factors like pH, temperature, surface area, functional groups, and the presence of other ions in the solution. These factors can affect the amount of energy required for adsorption, the availability of active sites for adsorption, and the competition for adsorption sites, which can all impact the adsorption capacity of the adsorbent. Thermodynamics parameters such as standard Gibb's free energy change (ΔG°), enthalpy change (ΔH°), and standard entropy change (ΔS^o^) were investigated for the operation of adsorption of P on BC-Zn adsorbent at different adsorption temperatures of 15, 20, 25, and 30 °C using Eqs. (8), (9), (10)) and results are shown in Table 4.

(8) $${\Delta G}^{o}=-RT\;\ln\;k_{d}$$

(9) $$\Delta G^{o}=\Delta H^{o}-T\Delta S^{o}$$

(10) $$\ln\;k_{d}=\frac{\Delta S^{o}}{R}-\frac{\Delta H^{o}}{RT}$$

where k_d_ represents the adsorption equilibrium constant (Q_e_/C_e_). R is the gas constant of 8.314 J.

**Table: 4:** Thermodynamic parameters on p adsorption onto BC-Zn adsorbent of adsorption isotherms model on P adsorption onto BC-Zn adsorbent.

Adsorbent	Temp. K	K_L_	ΔG^o^ (KJ mol^−1^)	ΔH^o^ (KJ mol^−1^)	ΔS^o^ (JK^−1^. mol^−1^)	R^2^
BC-Zn	288	0.180	4.095	−0.405	−25.593	0.999
	293	0.188	4.070			
	298	0.230	3.641			
	303	0.226	3.738			

mol^−1^ K^−1^. T is the reaction temperature (K). ΔH° and ΔS° values can be calculated from the slope

and intercept of the plot of lnk_d_ against T, respectively.

Adsorption endothermic and exothermic nature during the adsorption of P onto the BC-Zn has been determined by the analysis of ΔH value. The negative value of ΔH = −0.405 was observed during the adsorption process, which indicates the lower heat required for the P adsorption on the adsorbent, and the reaction was endothermic in nature. The disorder was calculated by the use of ΔS, where the negative values of △S^o^ indicated the decreased randomness during the P adsorption process. The adsorption process, such as spontaneous and non-spontaneous, are calculated using the ΔG value of the process with the change in the temperature. Gibb's free energy change, ΔG° at different temperatures viz., 15, 20, 25, and 30 °C are 4.095, 4.070, 3.641, and 3.738 kJ/mol, respectively. Positive ΔG° values indicate that the process of P adsorption onto BC-Zn adsorbent is not a rapid and non-spontaneous process. This result also matched properly with the reaction kinetics model.

### 3.3 P desorption study

The desorption or releasing rate of P after the adsorption of P onto the adsorbent is very important for the application in the soil or the recyclability of the adsorbent. Here, we carried out a desorption study at different times, such as 3, 18, and 24 h using distilled water (Figure S6). Results showed that with the increase in time, the desorption rate increases rapidly from 0.97% for 3 h to 97.08% for 24 h. It is indicated that Zn metal biochar could be suitable for the lower nutrient soil or recyclability for P adsorption. A high P fertilization rate in order to create a high soil P availability may increase the risk of P loss through soil leaching and groundwater contamination if erosion occurs. Moreover, due to the low availability of P and N in biochar could be inefficient for soil fertilizer. Thus, an appropriate nutrient amount should have maintained for the soil fertilizer application. Further adsorption of P onto recycled Zn-biochar adsorbent might be efficient for the removal of P from wastewater. Thus, Zn-biochar is a good method for adsorption because it can increase the biochar's surface area, add functional groups, decrease its pH sensitivity, is relatively inexpensive, and can be reusable. These properties can make activated biochar a more efficient and cost-effective solution for removing phosphorus from wastewater.

### 3.4 Phosphorus adsorption mechanism

**Fig. 7:**
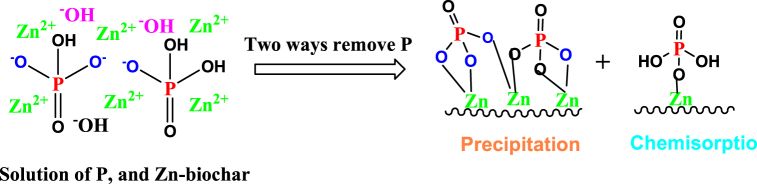
Adsorption mechanism of BC-Zn adsorbent.

Different characterization methods such as FT-IR, XRD, and XPS have been employed to understate the P adsorption mechanism onto the ZnCl_2_-activated biochar (BC–Zn). The phosphorus removal efficiency is depended on the chemical composition and nature of adsorbent materials, also the various operating conditions such as pH, contact time, initial phosphate concentration, and temperature. The adsorption process could be involved complex mechanisms with the different molecular interactions between the adsorbent and adsorbate, which consist of chemical and physical interaction. Higher adsorption of P was mainly due to the complexion and precipitation reaction between the HPO_4_^2−^ and Zn^2+,^ which could form a crystal of ZnHPO_4_ and Zn_5_(OH)(PO_4_)_3_ (Fig. 7). The chemical precipitation of P on the BC-Zn was confirmed by XPS analysis (Fig. 2). The P adsorbent surface was found to have Zn–P/Zn–P–OH based on the Zn2p spectrum. The O1s spectrum showed absorption peaks at 531.71 eV and 532.22 eV, which correspond to C=O/P=O and Zn–O, respectively, and at 133.87 eV and 134.82 eV, which represent Zn–P=O and Zn–P–OH. The binding energy at 1020.5–1100.10 eV was determined to be ZnHPO_4_. In addition, the stretching vibrations of P–O and P=O bonds in the BC-Zn-P material were identified by two FT-IR peaks at 853 cm^−1^ and 776 cm^−1^ (Fig. 1) [26]. Stronger adsorption peaks of modified biochar indicate that BC-Zn successfully adsorbs the P on it by the chemical interaction or complex precipitation reaction between the HPO_4_^2−^ and Zn^2+^. XRD analysis of before and after adsorption adsorbent revealed that after P adsorption biochar (BC–Zn–P) peaks slightly shifted to 9.5° compared to the BC-Zn adsorbent (Figure S3). It is due to the phase change of Zn metal after P adsorption by the chemical complex reaction. Further, peaks at 19.5°, 31.2°, 45.3°^,^ and 46.8° of the Zn–P, and Zn–O/Zn–OH phase confirmed that the process mainly occurred through the chemical complex formation reaction. Overall reaction mechanism concludes that the phosphate present at lower pH, such as in the form of H_3_PO_4_, and H_2_PO_4_^−^ are not capable of participating in chemical complex reactions with the Zn^2+^, while it occurred at higher pH between the phosphate form of HPO_4_^2−^ and Zn^2+^.

## 4 Conclusions

Pinewood biochar was modified using different chemical and metal-activated methods for P adsorption. ZnCl_2_ metal salt-modified biochar (BC–Zn) showed higher removal efficiency (100%). The kinetics study illustrated that the pseudo-second-order model provides a better explanation for the P adsorption on BC-Zn biochar. Langmuir isotherm adsorption capacity value (8.9 mg/g) is comparable to the experimental value (10.4 mg/g). This indicates that monolayer adsorption mainly occurred during the P adsorption onto the BC-Zn adsorbent. P desorption analysis showed that the desorption rate increased rapidly from 0.97% for 3 h to 97.08% for 24 h. P adsorption from a real wastewater study showed that BC-Zn adsorbent was able to adsorb 100% of the P from the wastewater. Hence, results suggested BC-Zn adsorbent to be a potential for P adsorption from real wastewater.

## Author contribution statement

Bijoy Biswas: Conceived and designed the experiments; Performed the experiments; Wrote the paper.

Tawsif Rahman, Manish Sakhakarmy: Performed the experiments; Analyzed and interpreted the data.

Hossein Jahromi, Mohamed Eisa: Analyzed and interpreted the data.

Jonas Baltrusaitis, Jasmeet Lamba, Allen Torbert: Contributed reagents, materials, analysis tools or data; Wrote the paper.

Sushil Adhikari: Conceived and designed the experiments; Wrote the paper.

## Data availability statement

Data will be made available on request.

## Declaration of competing interest

The authors declare that they have no known competing financial interests or personal relationships that could have appeared to influence the work reported in this paper.
