# Supplementary material for: Phosphorus adsorption using chemical and metal chloride activated biochars: Isotherms, kinetics and mechanism study
Source: Heliyon. 2023 Sep 4;9(9):e19830. doi: 10.1016/j.heliyon.2023.e19830 (PMC10559209; doi:10.1016/j.heliyon.2023.e19830)
Supplement: Multimedia component 1 [file mmc1.docx]

**Phosphorus adsorption using chemical and metal chloride activated biochars: Isotherms, kinetics and mechanism study**

Bijoy Biswas^a,b^, Tawsif Rahman^a^, Manish Sakhakarmy^a^, Hossein Jahromi^a,b^, Mohamed Eisa,^c^ Jonas Baltrusaitis^c^, Jasmeet Lamba^a^, Allen Torbert^d^, Sushil Adhikari^a,b*^

^a^Biosystems Engineering Department, 200 Corley Building, Auburn University, Auburn, AL 36849, USA

^b^Center for Bioenergy and Bioproducts, 519 Devall Drive, Auburn University, Auburn, AL 36849, USA

^c^Department of Chemical and Biomolecular Engineering, Lehigh University, USA

^d^National Soil Dynamics Laboratory, United States Department of Agriculture-Agriculture Research Service, Auburn, AL 36832, USA

*Corresponding author's e-mail: sushil.adhikari@auburn.edu (S. Adhikari)

**Supplementary Information**


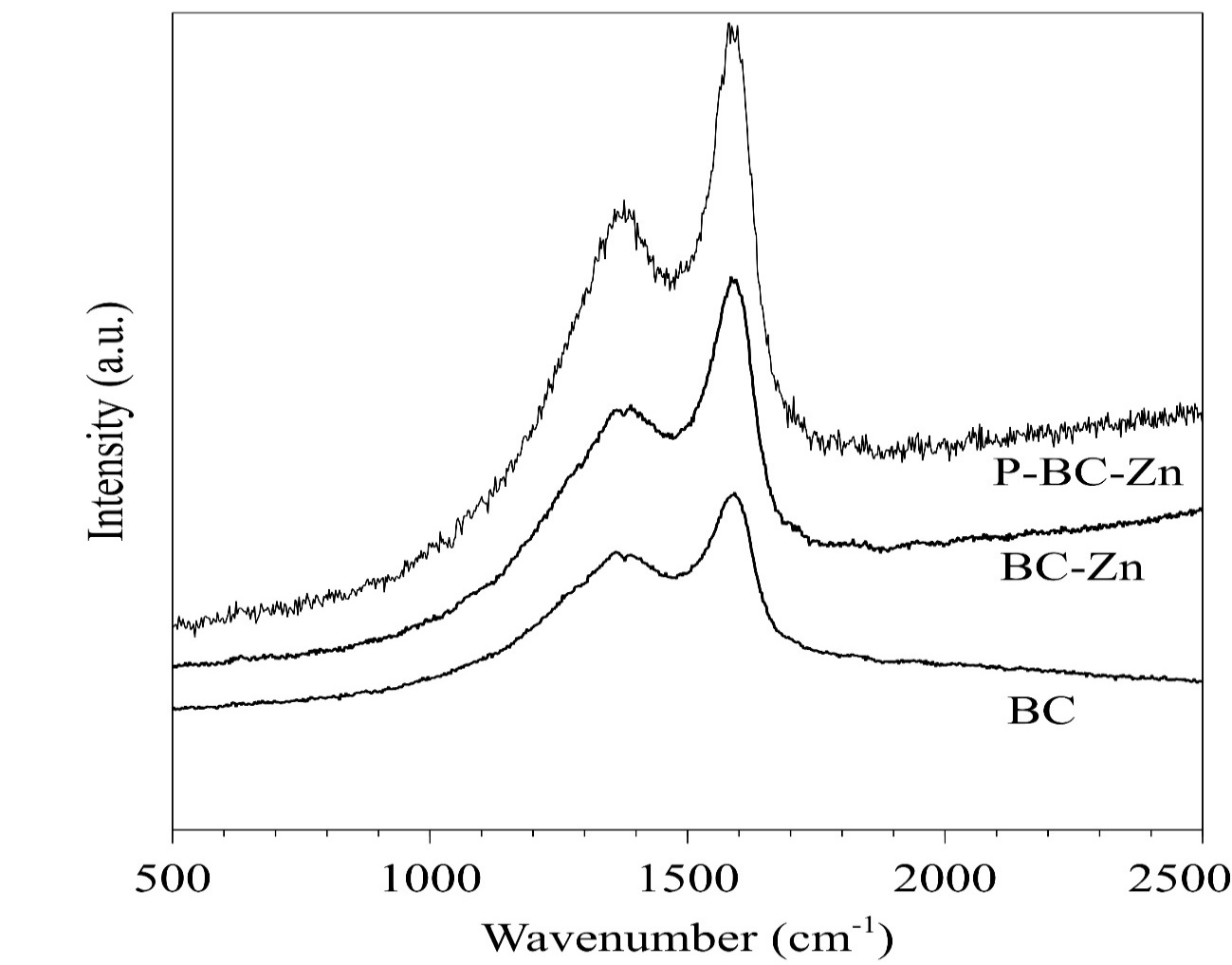


Figure S1. Raman spectra of raw bio-char and modified bio-chars

Figure S2: XRD analysis of different bio-char and after P adsorption biochar

Figure S3: Thermal stability analysis of modified biochar and after P adsorption biochar

Figure S4: Point of zero charge analysis of BC-Zn modified bio-char

Figure S5: Effect of Co-existing element present in solution on P removal rate

Figure S6: P desorption analysis at different time
